# Early management of patients with aneurysmal subarachnoid hemorrhage in a hospital with neurosurgical/neuroendovascular facilities: a consensus and clinical recommendations of the Italian Society of Anesthesia and Intensive Care (SIAARTI)—part 2

**DOI:** 10.1186/s44158-022-00049-4

**Published:** 2022-05-19

**Authors:** Edoardo Picetti, Andrea Barbanera, Claudio Bernucci, Alessandro Bertuccio, Federico Bilotta, Edoardo Pietro Boccardi, Tullio Cafiero, Anselmo Caricato, Carlo Alberto Castioni, Marco Cenzato, Arturo Chieregato, Giuseppe Citerio, Paolo Gritti, Luigi Lanterna, Roberto Menozzi, Marina Munari, Pietro Panni, Sandra Rossi, Nino Stocchetti, Carmelo Sturiale, Tommaso Zoerle, Gianluigi Zona, Frank Rasulo, Chiara Robba

**Affiliations:** 1 Department of Anesthesia and Intensive Care, Azienda Ospedaliero-Universitaria di Parma, Parma, Italy; 2 Department of Neurosurgery, “SS Antonio e Biagio e Cesare Arrigo” Hospital, Alessandria, Italy; 3 Department of Neuroscience and Surgery of the Nervous System, ASST Papa Giovanni XXIII Hospital, Bergamo, Italy; 4 Department of Anesthesiology and Critical Care, Policlinico Umberto I Hospital, La Sapienza University of Rome, Rome, Italy; 5 Department of Interventional Neuroradiology, ASST Grande Ospedale Metropolitano Niguarda, Milan, Italy; 6 Department of Anesthesia and Intensive Care Unit, AORN Cardarelli, Naples, Italy; 7 Department of Anesthesia and Critical Care, IRCCS A. Gemelli University Polyclinic Foundation, Rome, Italy; 8 Department of Anesthesia and Intensive Care, IRCCS Institute of Neurological Sciences of Bologna, Bologna, Italy; 9 Department of Neurosurgery, ASST Grande Ospedale Metropolitano Niguarda, Milan, Italy; 10 Neurointensive Care Unit, Department of Neuroscience and Department of Anesthesiology, ASST Grande Ospedale Metropolitano Niguarda, Milan, Italy; 11 School of Medicine and Surgery, University Milano – Bicocca, Milan, Italy; 12 Department of Anesthesia and Critical Care Medicine, Papa Giovanni XXIII Hospital, Bergamo, Italy; 13 Interventional Neuroradiology Unit, University Hospital of Parma, Parma, Italy; 14 Anesthesia and Intensive Care, Padua University Hospital, Padua, Italy; 15 Department of Neuroradiology, San Raffaele Hospital, Milan, Italy; 16 Neuroscience Intensive Care Unit, Department of Anesthesia and Critical Care, Fondazione IRCCS Ca’ Granda Ospedale Maggiore Policlinico, Milan, Italy; 17 Department of Pathophysiology and Transplantation, University of Milan, Milan, Italy; 18 Neurosurgery Unit, IRCCS Istituto delle Scienze Neurologiche Ospedale Bellaria di Bologna, Bologna, Italy; 19 Department of Neurosurgery, Policlinico San Martino Hospital, IRCCS for Oncology and Neuroscience, Genoa, Italy; 20 Department of Anesthesia, Intensive Care and Emergency Medicine, Spedali Civili University Hospital, Brescia, Italy; 21 Anesthesia and Intensive Care, San Martino Policlinico Hospital, IRCCS for Oncology and Neurosciences, Genoa, Italy; 22 Department of Surgical Sciences and Integrated Diagnostics, University of Genoa, Genoa, Italy

**Keywords:** Subarachnoid hemorrhage, Vasospasm, Delayed cerebral ischemia, Monitoring, Intracranial hypertension

## Abstract

**Background:**

Questions remain on the optimal management of subarachnoid hemorrhage (SAH) patients once they are admitted to the referring center, before and after the aneurysm treatment. To address these issues, we created a consensus of experts endorsed by the Italian Society of Anesthesia and Intensive Care (SIAARTI) to provide clinical guidance regarding this topic. Specifically, in this manuscript (part 2), we aim to provide a list of experts’ recommendations regarding the management of SAH patients in a center with neurosurgical/neuroendovascular facilities after aneurysm treatment.

**Methods:**

A multidisciplinary consensus panel composed by 24 physicians selected for their established clinical and scientific expertise in the acute management of SAH patients with different specializations (anesthesia/intensive care, neurosurgery, and interventional neuroradiology) was created. A modified Delphi approach was adopted.

**Results:**

A total of 33 statements were discussed, voted, and approved. Consensus was reached on 30 recommendations (28 strong and 2 weak). In 3 cases, where consensus could not be agreed upon, no recommendation was provided.

**Conclusions:**

This consensus provides practical recommendations (and not mandatory standard of practice) to support clinician’s decision-making in the management of SAH patients in centers with neurosurgical/neuroendovascular facilities after aneurysm securing.

## Background

Aneurysmal subarachnoid hemorrhage (SAH) is a complex and multifaceted pathology which plays out over days to weeks and which often requires prolonged intensive care unit (ICU) stay [1, 2]. Initial care of aneurysmal SAH patients is aimed at stabilizing life-threatening conditions, particularly for comatose patients with impaired respiratory and hemodynamic function [1–3]. Despite early aggressive resuscitation and multidisciplinary ICU management have shown to be potentially associated with improved outcomes [4], the mortality remains high and the complication rate of these patients can be also related to factors occurring after initial stabilization and aneurysm treatment [5].

Common problems in this phase include systemic factors (i.e., fever, hyperglycemia, hyponatremia, cardiopulmonary dysfunction, infections, etc.) as well as SAH-specific complications such as vasospasm and delayed cerebral infarction [6, 7].

The management of SAH patients focuses on the anticipation, prevention, and management of these secondary complications and thus can be particularly challenging for the intensivist [3, 7]. However, a high level of evidence is lacking even regarding the management of SAH in this phase.

Therefore, the Italian Society of Anesthesia, Intensive Care and Pain Medicine (SIAARTI) endorsed and supported the creation of an expert consensus with the aim to provide clinical recommendations and help the clinicians in the pragmatic approach of SAH patients. This manuscript represents part 2 of a Delphi process specifically focusing on the management of SAH after aneurysm securing and therefore on late complications and management of these patients.

## Methods

The methodology of the consensus has been previously described in detail [8, 9]. Briefly, the project was commissioned and approved by the Executive Committee of the SIAARTI which supported and supervised the development and the methodology of the consensus.

A steering committee of researchers/clinicians and a non-voting methodologist, which included EP, CR, and FR, was defined in accordance with the SIAARTI and was in charge for the selection of the experts’ panel. Criteria for inclusion were neuroanesthetists and neurointensivists (all SIAARTI members), with established clinical expertise in the management of SAH, and a representation of neurosurgeons and neuroradiologists. The steering committee also defined the timeline, aims, and methodology, engaged with the research group of the SIAARTI for the development of the consensus though serial teleconferences from August 2021 to February 2022.

For this part of the consensus, following a non-systematic review regarding the clinical management of SAH patients after aneurysm treatment (coiling or clipping), the panel and the steering committee identified the domains and generated a list of questions which underwent a Delphi process after the panel approval [10, 11]. Three online surveys were distributed to the panel, and experts were asked to express their degree of agreement possibility adding specific comments during the first two rounds, which were used to modify/improve the statements. A *strong recommendation* was defined for a threshold of agreement >85%, *weak recommendation* for 75–85% agreement, and *no recommendation for* < 75% after the rounds*.*

## Results

**Fig. 1:**
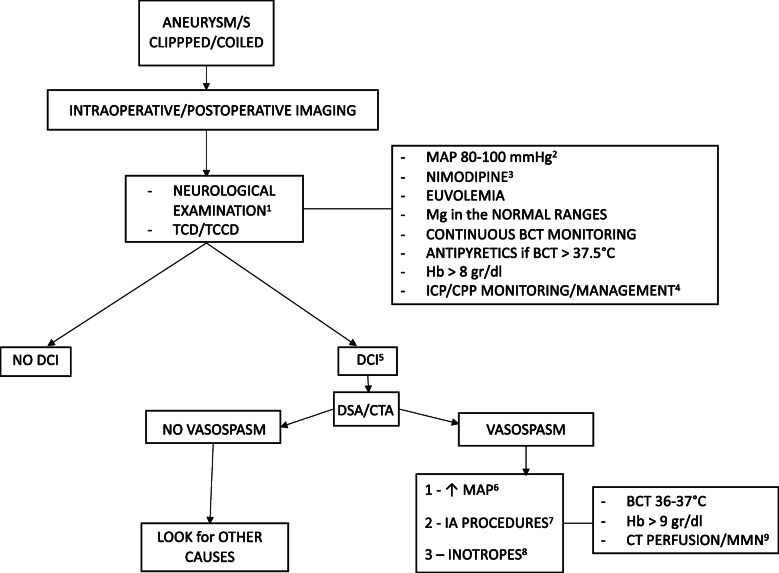
Consensus flow chart. ^1^Excluding confounders such as sedation, hypo/hyperthermia, hypoxia, and hypercapnia are advisable. Contraindications to sedation hold can be only ICP instability, radiological signs of intracranial hypertension, and severe respiratory failure. ^2^Close to the lower limit (80 mmHg) for patients without a history of arterial hypertension and close to the upper (100 mmHg) for patients with a history of arterial hypertension. ^3^60 mg every 4 h for 21 days after bleeding (oral or nasogastric tube); intravenous nimodipine (2 mg/h) in case of feeding intolerance. Do not utilize in hemodynamically unstable SAH patients or withheld in case of a significant drop in arterial blood pressure. ^4^ICP monitoring in all salvageable SAH patients in coma (GCS ≤ 8) with radiological signs of intracranial. Elevated ICP management (aneurysm/s secured) should take into account the underlying pathophysiological mechanism responsible of intracranial hypertension. CPP ≧ 70 mmHg. ^5^Regarding neurological examination, we consider as suggestive of DCI-related vasospasm the occurrence of a new focal or a global neurologic deficit or a decrease of 2 or more points on the GCS score that lasts for at least 1 h and cannot be explained by another cause. Regarding TCD/TCCD examination, we consider as suggestive of vasospasm an increase in mean FVMCA of more than 50 cm/s from basal over 24 h and/or a mean FVMCA of at least 120 cm/s (with a suggestive Lindegaard ratio). ^6^Up to 120 mmHg and targeted to the resolution of clinical symptoms and/or radiological findings taking into account the patient’s cardiovascular status to minimize the risks associated with MAP augmentation. ^7^Vasodilators and/or angioplasty. The treatment should be individualized after discussion with the interventional neuroradiologist. ^8^With advanced hemodynamic monitoring. ^9^If available, to utilize therapy personalization for DCI associated with cerebral vasospasm in poor-grade SAH patients where neurological assessment is not possible. Abbreviations: TCD transcranial doppler, TCCD transcranial color doppler, MAP mean arterial pressure, Mg magnesium, BCT body core temperature, Hb hemoglobin, ICP intracranial pressure, CPP cerebral perfusion pressure, DCI delayed cerebral ischemia, CTA computed tomography angiography, DSA digital subtraction angiography, IA intra-arterial, CT computed tomography, MMN multimodal neuromonitoring

The second part of this consensus included 33 statements and provided 30 recommendations (Table 1): 28 were strong recommendations, endorsed by more than 85% of participants, while 2 were weak recommendations, supported by 75–85%. The consensus flow chart is reported in Fig. 1. We were unable to reach consensus for 3 statements. The consensus recommendations are listed below with the percentage of agreement.

**Table 1:** List of consensus recommendations

N.	Recommendation	Level
1	We recommend the utilization of intraoperative (if available) and postoperative imaging to verify the correct management of the aneurysm/s and to exclude cerebral ischemia/bleeding related to the procedure (agreement 95.5%, strong recommendation).	*Strong recommendation*
2	We recommend, as soon as possible after cerebral aneurysm/s securing, the assessment of the neurological status excluding confounders such as sedation, hypo/hyperthermia, hypoxia, and hypercapnia. Contraindications to sedation hold can be only ICP instability, radiological signs of intracranial hypertension, and severe respiratory failure.	*Strong recommendation*
3	We recommend to monitor the patient with repeated neurological and/or TCD/TCCD examinations to raise the suspect of DCI associated with cerebral vasospasm.	*Strong recommendation*
4	We recommend, regarding neurological examination, to consider as suggestive of DCI-related vasospasm, the occurrence of a new focal or a global neurologic deficit or a decrease of 2 or more points on the GCS score that lasts for at least 1 h and cannot be explained by another cause.	*Strong recommendation*
5	We recommend, regarding TCD/TCCD examination, to consider signs suggestive of vasospasm: an increase in mean FVMCA of more than 50 cm/s from basal over 24 h and/or a mean FVMCA of at least 120 cm/s (with a suggestive Lindegaard ratio).	*Strong recommendation*
6	We recommend the utilization of CTA and/or DSA to confirm the presence of cerebral vasospasm as the cause of the DCI in case of neurological examination or TCD/TCCD suggestive for vasospasm.	*Strong recommendation*
7	We recommend, in SAH patients with DCI related to cerebral vasospasm, the utilization of advanced perfusion imaging (i.e., CT perfusion, MRI perfusion) to early assess the development of ischemic brain lesions.	*Strong recommendation*
8	We recommend, after cerebral aneurysm/s treatment, in patients without intracranial hypertension and vasospasm, the maintenance of a MAP between 80 and 100 mmHg.	*Strong recommendation*
9	We recommend the maintenance of MAP values close to the lower limit (80 mmHg) for patients without a history of arterial hypertension.	*Strong recommendation*
10	We recommend the maintenance of MAP values close to the upper limit (100 mmHg) for patients with a history of arterial hypertension.	*Strong recommendation*
11	We recommend the maintenance of a CPP ≧ 70 mmHg in patients with intracranial hypertension.	*Strong recommendation*
12	We recommend the maintenance of euvolemia in all salvageable poor-grade SAH patients.	*Strong recommendation*
13	We recommend that oral nimodipine (60 mg every 4 h) be administered (as the first choice) for 21 days after bleeding for DCI prevention.	*Strong recommendation*
14	We recommend the administration of intravenous nimodipine (2 mg/h) in case of feeding intolerance.	*Weak recommendation*
15	We recommend against the administration of oral/intravenous nimodipine in hemodynamically unstable SAH patients (i.e., under inotropes and/or vasopressors therapy).	*Weak recommendation*
16	We recommend to withhold oral/intravenous nimodipine in case of a significant drop in arterial blood pressure (see recommendation 8).	*Strong recommendation*
17	We recommend the maintenance of a magnesium blood level in the normal ranges in all SAH patients for 21 days after bleeding for vasospasm prevention.	*Strong recommendation*
18	We recommend the maintenance of a Hb level > 8 gr/dl in poor-grade SAH patients without DCI-related vasospasm.	*Strong recommendation*
19	We recommend a continuous BCT monitoring in poor-grade SAH patients.	*Strong recommendation*
20	Being fever (regardless of the cause that needs to be investigated) associated with poor outcome after SAH, we recommend the administration of antipyretics for a BCT > 37.5 in poor-grade SAH patients without DCI-related vasospasm.	*Strong recommendation*
21	We recommend ICP monitoring in all salvageable SAH patients in coma (GCS ≤ 8) with radiological signs of intracranial hypertension.	*Strong recommendation*
22	We recommend the management of elevated ICP in all salvageable SAH patients (aneurysm/s secured) taking into account the underlying pathophysiological mechanism responsible of intracranial hypertension.	*Strong recommendation*
23	We recommend, in case of DCI associated with cerebral vasospasm (symptomatic vasospasm), hemodynamic optimization increasing arterial blood pressure as first step of treatment.	*Strong recommendation*
24	We recommend that hemodynamic optimization (i.e., gradual increase of MAP) should be targeted to the resolution of clinical symptoms and/or radiological findings. This process should take into account the patient’s cardiovascular status to minimize the risks associated with MAP augmentation.	*Strong recommendation*
25	We recommend, in case of DCI associated with cerebral vasospasm (symptomatic vasospasm) refractory to an increase in arterial blood pressure (MAP not greater than 120 mmHg), the utilization of invasive intra-arterial procedures.	*Strong recommendation*
26	We recommend that the choice of intra-arterial procedure (i.e., vasodilators, angioplasty) be individualized after discussion with the interventional neuroradiologist (see recommendation 25).	*Strong recommendation*
27	We recommend the maintenance of an Hb level > 9 gr/dl in case of DCI associated with cerebral vasospasm.	*Strong recommendation*
28	We recommend the maintenance of normothermia (a BCT between 36 and 37 °C) in case of DCI associated with cerebral vasospasm.	*Strong recommendation*
29	We recommend that CT perfusion and/or advanced multimodal neuromonitoring (i.e., brain tissue oxygenation monitoring, etc.), if available, be utilized to guide (individualize) therapy for DCI associated with cerebral vasospasm in poor-grade SAH patients where neurological assessment is not possible.	*Strong recommendation*
30	We recommend the utilization of inotropes for refractory vasospasm with the utilization of an advanced hemodynamic monitoring.	*Strong recommendation*
31	We are unable to provide any recommendation regarding the utilization of therapeutic hypothermia in case of DCI related to refractory vasospasm.	*No recommendation*
32	We are unable to provide any recommendation regarding the utilization of metabolic suppression in case of DCI related to refractory vasospasm.	*No recommendation*
33	We are unable to provide any recommendation regarding the utilization of milrinone in case of DCI related to refractory vasospasm.	*No recommendation*

*Abbreviations*: *ICP* intracranial pressure, *TCD* transcranial Doppler, *TCCD* transcranial color Doppler, *DCI* delayed cerebral ischemia, *GCS* Glasgow coma scale, *FVMCA* flow velocity mean cerebral artery, *SAH* subarachnoid hemorrhage, *CT* computed tomography, *CTA* CT angiography, *DSA* digital subtraction angiography, *CPP* cerebral perfusion pressure, *MRI* magnetic resonance imaging, *MAP* mean arterial pressure, *Hb* hemoglobin, *BCT* body core temperature

### Recommendation 1

We recommend the utilization of intraoperative (if available) and postoperative imaging to verify the correct management of the aneurysm/s and to exclude cerebral ischemia/bleeding related to the procedure (agreement 95.5%, strong recommendation).

### Recommendation 2

We recommend, as soon as possible after cerebral aneurysm/s securing, the assessment of the neurological status excluding confounders such as sedation, hypo/hyperthermia, hypoxia, and hypercapnia. Contraindications to sedation hold can be intracranial pressure (ICP) instability, radiological signs of intracranial hypertension, and severe respiratory failure (agreement 86.5%, strong recommendation).

### Recommendation 3

We recommend to monitor the patient with repeated neurological and/or transcranial Doppler (TCD)/transcranial color Doppler (TCCD) examinations to raise the suspect of delayed cerebral ischemia (DCI) associated with cerebral vasospasm (agreement 86.5%, strong recommendation).

### Recommendation 4

We recommend, regarding neurological examination, to consider as suggestive of DCI-related vasospasm, the occurrence of a new focal or a global neurologic deficit or a decrease of 2 or more points on the Glasgow Coma Scale (GCS) score that lasts for at least 1 h and cannot be explained by another cause (agreement 95.5%, strong recommendation).

### Recommendation 5

We recommend, regarding TCD/TCCD examination, to consider signs suggestive of vasospasm: an increase in mean flow velocity in the middle cerebral artery (FVMCA) of more than 50 cm/s from basal over 24 h and/or a mean FVMCA of at least 120 cm/s (with a suggestive Lindegaard ratio) (agreement 91%, strong recommendation).

### Recommendation 6

We recommend the utilization of computed tomography angiography (CTA) and/or digital subtraction angiography (DSA) to confirm the presence of cerebral vasospasm as the cause of the DCI in case of neurological examination or TCD/TCCD suggestive for vasospasm (agreement 86.5%, strong recommendation).

### Recommendation 7

We recommend, in SAH patients with DCI related to cerebral vasospasm, the utilization of advanced perfusion imaging [i.e., CT perfusion, magnetic resonance imaging (MRI) perfusion] to early assess the development of ischemic brain lesions (agreement 100%, strong recommendation).

### Recommendation 8

We recommend, after cerebral aneurysm/s treatment, in patients without intracranial hypertension and vasospasm, the maintenance of a mean arterial pressure (MAP) between 80 and 100 mmHg (agreement 86.5%, strong recommendation).

### Recommendation 9

We recommend the maintenance of MAP values close to the lower limit (80 mmHg) for patients without a history of arterial hypertension (agreement 86.5%, strong recommendation).

### Recommendation 10

We recommend the maintenance of MAP values close to the upper limit (100 mmHg) for patients with a history of arterial hypertension (agreement 91%, strong recommendation).

### Recommendation 11

We recommend the maintenance of a cerebral perfusion pressure (CPP) ≧ 70 mmHg in patients with intracranial hypertension (agreement 95.5%, strong recommendation).

### Recommendation 12

We recommend the maintenance of euvolemia in all salvageable poor-grade SAH patients (agreement 95.5%, strong recommendation).

### Recommendation 13

We recommend that oral nimodipine (60 mg every 4 h) be administered (as the first choice) for 21 days after bleeding for DCI prevention (agreement 100%, strong recommendation).

### Recommendation 14

We recommend the administration of intravenous nimodipine (2 mg/h) in case of feeding intolerance (see recommendation 13) (agreement 81%, weak recommendation).

### Recommendation 15

We recommend against the administration of oral/intravenous nimodipine in hemodynamically unstable SAH patients (i.e., under inotropes and/or vasopressors therapy) (agreement 77%, weak recommendation).

### Recommendation 16

We recommend to withheld oral/intravenous nimodipine in case of a significant drop in arterial blood pressure (see recommendation 8) (agreement 95.5%, strong recommendation).

### Recommendation 17

We recommend the maintenance of a magnesium blood level in the normal ranges in all SAH patients for 21 days after bleeding for vasospasm prevention (agreement 100%, strong recommendation).

### Recommendation 18

We recommend the maintenance of a hemoglobin (Hb) level > 8 gr/dl in poor-grade SAH patients without DCI-related vasospasm (agreement 91%, strong recommendation).

### Recommendation 19

We recommend a continuous body core temperature (BCT) monitoring in poor-grade SAH patients (agreement 95.5%, strong recommendation).

### Recommendation 20

Being fever (regardless of the cause that needs to be investigated) associated with poor outcome after SAH, we recommend the administration of antipyretics for a BCT > 37.5 in poor-grade SAH patients without DCI-related vasospasm (agreement 95.5%, strong recommendation).

### Recommendation 21

We recommend ICP monitoring in all salvageable SAH patients in coma (GCS ≤ 8) with radiological signs of intracranial hypertension (agreement 95.5%, strong recommendation).

### Recommendation 22

We recommend the management of elevated ICP in all salvageable SAH patients (aneurysm/s secured) taking into account the underlying pathophysiological mechanism responsible of intracranial hypertension (agreement 100%, strong recommendation).

### Recommendation 23

We recommend, in case of DCI associated with cerebral vasospasm (symptomatic vasospasm), hemodynamic optimization increasing arterial blood pressure as first step of treatment (agreement 95.5%, strong recommendation).

### Recommendation 24

We recommend that hemodynamic optimization (i.e., gradual increase of MAP) should be targeted to the resolution of clinical symptoms and/or radiological findings. This process should take into account the patient’s cardiovascular status to minimize the risks associated with MAP augmentation (agreement 95.5%, strong recommendation).

### Recommendation 25

We recommend, in case of DCI associated with cerebral vasospasm (symptomatic vasospasm) refractory to an increase in arterial blood pressure (MAP not greater than 120 mmHg), the utilization of invasive intra-arterial procedures (agreement 95.5%, strong recommendation).

### Recommendation 26

We recommend that the choice of intra-arterial procedure (i.e., vasodilators, angioplasty) be individualized after discussion with the interventional neuroradiologist (see recommendation 25) (agreement 95.5%, strong recommendation).

### Recommendation 27

We recommend the maintenance of an Hb level > 9 gr/dl in case of DCI associated with cerebral vasospasm (agreement 91%, strong recommendation).

### Recommendation 28

We recommend the maintenance of normothermia (a BCT between 36 and 37 °C) in case of DCI associated with cerebral vasospasm (agreement 95.5%, strong recommendation).

### Recommendation 29

We recommend that CT perfusion and/or advanced multimodal neuromonitoring (i.e., brain tissue oxygenation monitoring, etc.), if available, be utilized to guide (individualize) therapy for DCI associated with cerebral vasospasm in poor-grade SAH patients where neurological assessment is not possible (agreement 91%, strong recommendation).

### Recommendation 30

We recommend the utilization of inotropes for refractory vasospasm with the utilization of an advanced hemodynamic monitoring (agreement 91%, strong recommendation).

### Recommendation 31

We are unable to provide any recommendation regarding the utilization of therapeutic hypothermia in case of DCI related to refractory vasospasm (agreement 50%, no recommendation).

### Recommendation 32

We are unable to provide any recommendation regarding the utilization of metabolic suppression in case of DCI related to refractory vasospasm (agreement 64%, no recommendation).

### Recommendation 33

We are unable to provide any recommendation regarding the utilization of milrinone in case of DCI related to refractory vasospasm (agreement 64%, no recommendation).

## Discussion

### Postoperative imaging

Perioperative imaging can be useful to detect complications during and/or immediately after surgical clipping [12, 13]. In particular, intraoperative angiography has proven to be useful in recognizing residual aneurysm and vessel flow compromise resulting in a rapid treatment [12, 13]. The most recent SAH guidelines of the American Heart Association (AHA) recommend, after any aneurysm repair, an immediate cerebrovascular imaging to identify remnants or recurrence of the aneurysm that may require treatment (class I; level of evidence B). We agree with this recommendation and, in addition, we believe that, as soon as possible after cerebral aneurysm/s securing, the assessment of the neurological status is advisable in the absence of contraindications to sedation hold such as ICP instability, radiological signs of intracranial hypertension, and severe respiratory failure.

### DCI-related vasospasm monitoring

DCI, especially if associated to vasospasm, is a major cause of death and disability after SAH [2, 7]. In this regard, a rapid diagnosis and treatment is of paramount importance to prevent cerebral infarction [2, 7]. Serial neurological examinations are fundamental to detect the occurrence of new ischemic insults — i.e., especially in awake patients or in those who may undergo a reliable neurological evaluation — but they are of limited sensitivity in patients with poor clinical grade [3, 5, 7]. Regarding neurological examination and according to available literature [14], we consider suggestive of DCI-related vasospasm the development of a new focal or a global neurologic deficit or a decrease of 2 or more points on the GCS score that lasts for at least 1 h and cannot be explained by another cause (i.e., seizures, hydrocephalus, etc.). Intermittent screening or continuous monitoring methods can be also useful for the detection and confirmation of DCI, especially in sedated or poor-grade SAH patients [5, 7, 14]. TCD/TCCD examination is commonly used as a noninvasive tool to monitor for the presence of cerebral vasospasm following acute SAH [3, 7]. TCD/TCCD has a good sensitivity and specificity to detect vasospasm in the proximal segments of the middle cerebral artery (MCA) and internal carotid artery (ICA) but is less reliable regarding anterior cerebral artery (ACA) branches and posterior circulation arteries [3, 7]. The Lindegaard ratio (the ratio of mean MCA flow velocity divided by mean ICA flow velocity) is generally utilized to diagnose vasospasm in the MCA when the ratio is greater than 3 [3, 7, 12]. However, the sensitivity and specificity of TCD for cerebral vasospasm detection is operator dependent and some patients do not have adequate temporal bone windows to allow the detection of TCD signals [3, 7, 12]. According to available literature [15], we consider suggestive of vasospasm an increase in mean FVMCA of more than 50 cm/s from basal over 24 h and/or a mean FVMCA of at least 120 cm/s (with a suggestive Lindegaard ratio). Considering the above, DSA is considered the gold standard for the detection of cerebral vasospasm [7, 16]. Also, CTA can be used in this setting [16]. We recommend the utilization of CTA and/or DSA to confirm the presence of cerebral vasospasm as the cause of the DCI in case of neurological signs or TCD/TCCD suggestive for vasospasm. Moreover, considering that perfusion imaging with CT or MRI can be useful to identify regions of potential brain ischemia [3, 5, 7, 12], we recommend their use early in SAH patients with DCI related to cerebral vasospasm. This evaluation could be very important to modulate the intensity of care according to the extent of brain damage. Despite their possible utility and likely important applications, we have not considered continuous monitoring methods (i.e., brain tissue oxygenation monitoring, continuous electroencephalogram, etc.) because at present they are not yet widely available.

### DCI-related vasospasm prophylaxis

Nimodipine, a dihydropyridine calcium channel antagonist blocking the flux of extracellular calcium via voltage-gated calcium channels, is the only therapeutic agent with class I evidence for decreasing the risk of poor outcome in SAH [3, 7]. The beneficial effects of nimodipine are related to the reduction of delayed cerebral infarction occurrence but no effect on cerebral vasospasm was detected in large clinical trials. Thus, different potential mechanisms were proposed such as reduction of calcium-dependent excitotoxicity and reduced platelet aggregation [17]. It should be administered orally or by nasogastric tube at a dose of 60 mg every 4 h for 21 days [16]. In case of feeding intolerance, nimodipine should be applied intravenously [18]. The dose needs to be reduced or discontinued in case of arterial hypotension. According to above, we recommend:

- Administration of oral nimodipine (60 mg every 4 h) after bleeding for DCI prevention (administration of intravenous nimodipine at 2 mg/h in case of feeding intolerance)
- No administration of oral/intravenous nimodipine in hemodynamically unstable SAH patients (i.e., under inotropes and/or vasopressors therapy)
- To withhold oral/intravenous nimodipine in case of a significant drop in arterial blood pressure

Regarding this last point, it must be considered that the optimal MAP target, in SAH patients without vasospasm and intracranial hypertension after cerebral aneurysm treatment, has yet to be established. An individualized approach for blood pressure management, especially in unconscious patients, could be performed with a multimodality neuromonitoring (brain tissue oxygenation monitoring, electroencephalography, invasive quantitative cerebral blood flow monitoring, cerebral microdialysis, and electrocorticography) [19]. Unfortunately, not all centers worldwide have this possibility. For this reason, we recommend to maintain SAH patients slightly hypertensive with the maintenance of a MAP between 80 and 100 mmHg (close to the lower limit for patients without a history of arterial hypertension and close to the upper limit for patients with a history of arterial hypertension).

Magnesium is a non-competitive calcium antagonist with several vascular and neuroprotective effects (i.e., vasodilation, reduction in glutamate release, etc.) [20]. At the moment, the induction of hypermagnesemia for the prevention of DCI-related vasospasm is not supported while hypomagnesemia should be avoided [7, 16]. In this regard, we recommend the maintenance of a magnesium blood level in the normal ranges (1.6–2.5 mg/dl to 0.65–1 mmol/L) in all SAH patients for 21 days after bleeding for vasospasm prevention.

### DCI-related vasospasm management

Despite DCI has a complex multifactorial pathogenesis, ischemia from vasospasm is one of the most important potentially clinically reversible factors [3, 19]. Vasospasm typically occurs between 3 and 14 days post-bleeding, although it can occasionally persist up to 21 days [3, 7]. Angiographic vasospasm may or may not be clinically symptomatic [3]. Precisely, some degrees of vasospasm are visible angiographically in up to 70% of patients with SAH but only 30% of all patients develop clinical symptoms (symptomatic vasospasm) [3]. Symptomatic vasospasm, being associated with DCI and poor outcome following SAH, requires a prompt intervention [3, 19]. Generally, the first step in case of DCI-related vasospasm is to increase arterial blood pressure maintaining the patient euvolemic [5, 16, 19]. In this case, the co-administration of oral/intravenous nimodipine is not advisable [16]. We recommend a gradual increase of MAP (up to 120 mmHg), maintaining euvolemia, aimed to the resolution of clinical symptoms and/or radiological findings. This process should take into account the patient’s cardiovascular status to minimize the risks associated with MAP augmentation. We recommend that CT perfusion and/or advanced multimodal neuromonitoring (i.e., brain tissue oxygenation monitoring, electroencephalography, invasive quantitative cerebral blood flow monitoring, cerebral microdialysis, and electrocorticography), if available, should be utilized to guide (individualize) therapy for DCI associated with cerebral vasospasm in poor-grade SAH patients where neurological assessment is not possible. This strategy is in agreement with the available literature [5, 16, 19].

Generally, a variety of invasive intra-arterial procedures (angioplasty, vasodilators) are utilized in case of vasospasm refractory to MAP augmentation [5, 16, 19, 21]. Percutaneous transluminal balloon angioplasty (PTCA), based on mechanical stretching/dilation of vasospastic arteries, can be utilized only in case of proximal vessel vasospasm (i.e., internal carotid artery, M1 segments of the MCA) [19, 21]. This type of treatment, with respect to vasodilators, has a high success rate and is long-lasting [19, 21]. However, PTCA can present serious complications including embolism, thrombosis, dissection, and vessel rupture [19, 21]. Over the years, numerous intra-arterial vasodilators have been evaluated such as papaverine, nicardipine, verapamil, nimodipine, and milrinone [7]. Possible advantages of intra-arterial vasodilators with respect to PTCA are a distal and more diffuse effect and a better safety profile [7–21]. In some cases (distal + proximal vessels vasospasm), they can be used in conjunction with PTCA [21]. Possible disadvantages of vasodilators are recurrent vasospasm due to the short-lasting effect of these agents, intracranial hypertension due to vasodilation, and arterial hypotension due to systemic effects [21]. Data demonstrating the superiority of one method of treatment with respect to another are not available and more studies are necessary. Considering the above, we recommend, in case of symptomatic vasospasm refractory to MAP augmentation, the utilization of invasive intra-arterial procedures. In our opinion, the choice of intra-arterial procedure — i.e., vasodilators, angioplasty — should be individualized after discussion with the interventional neuroradiologist.

Increasing cardiac output with inotropes (i.e., dobutamine, milrinone) can improve brain perfusion after SAH [21]. A trial of inotropic therapy has been suggested if DCI-related vasospasm does not improve with blood pressure augmentation [16]. In this case, the utilization of an advanced hemodynamic monitoring (i.e., arterial pulse contour/waveform analysis, pulmonary artery catheter, transpulmonary thermodilution, ultrasound, etc.) was generally utilized [21]. We recommend the utilization of inotropes for refractory vasospasm with the utilization of an advanced hemodynamic monitoring. Milrinone, possessing a mechanism of action for the reversal of cerebral vasospasm as well as potentially anti-inflammatory effects, has been identified as a promising therapeutic agent for DCI [22]. Several recent preliminary studies showed a potential benefit of milrinone for the management of DCI-related vasospasm and encourage the conduction of confirmatory randomized trials [23–25]. Awaiting more data from well-powered studies, we are unable to provide any recommendation regarding the utilization of milrinone in case of DCI related to refractory vasospasm.

Anemia, reported in more than 50% of SAH patients, is associated with poor outcome [3, 5, 16, 21]. The appropriate target Hb concentration in SAH patients with and without DCI is unknown [3, 16]. Hb levels < 9g/dl are associated with brain tissue hypoxia and metabolic distress in poor-grade patients [26]. Packed red blood cell (RBC) transfusion increases brain tissue oxygen tension in poor-grade SAH patients with a baseline hemoglobin level of 8 g/dL [27]. RBC transfusion seems to be useful to optimize cerebral oxygen delivery in case of brain ischemia related to vasospasm. According to Neurocritical Care Society (NCS) guidelines, patients should receive packed RBC transfusions to maintain Hb concentration above 8–10 g/dl. Awaiting more data from well-powered studies, we recommend the maintenance of a Hb level > 8 gr/dl in poor-grade SAH patients without DCI-related vasospasm and > 9 gr/dl in case of DCI associated with cerebral vasospasm.

Fever is associated with a high rate of DCI and unfavorable outcomes after SAH [3, 28]. Temperature should be monitored frequently and infectious causes of fever should always be investigated and treated [16]. An aggressive control of fever, especially during the period of risk for DCI, is advisable [5, 16]. The intensity of fever control should be proportional to the risk of cerebral ischemia [16]. We recommend a continuous BCT monitoring in poor-grade SAH patients. Being fever (regardless of the cause that needs to be investigated) associated with poor outcome after SAH, we recommend the administration of antipyretics for a BCT > 37.5 in poor-grade SAH patients without DCI-related vasospasm and the maintenance of normothermia (BCT 36–37 °C) in case of DCI associated with cerebral vasospasm. Therapeutic hypothermia has been suggested as a possible rescue therapy for refractory symptomatic vasospasm [21], but considering their potential side effects [29] and the lack of robust data in this setting, we are unable to provide any recommendation regarding the utilization of therapeutic hypothermia in case of DCI related to refractory vasospasm. The panel expressed the same concerns for the utilization of metabolic suppression.

### ICP monitoring and intracranial hypertension management

Intracranial hypertension after SAH is associated with poor outcomes [30–32]. Existing SAH international guidelines [5, 16, 18] do not provide specific recommendations regarding ICP/CPP monitoring and treatment despite there being several reasons for doing so [33]. Considering the above, we recommend:

- ICP monitoring in all salvageable SAH patients in coma (GCS ≤ 8) with radiological signs of intracranial hypertension
- The management of elevated ICP in all salvageable SAH patients (aneurysm/s secured) taking into account the underlying pathophysiological mechanism responsible of intracranial hypertension
- The maintenance of a CPP ≧ 70 mmHg in patients with intracranial hypertension

### Limitations

This paper, similarly to the first part [9], has several limitations. We deliberately did not base our statements on systematic literature reviews because of the lack of evidence (previously underlined) and in favor of simple, basic topics that are rarely subject to investigation. We decided to produce the clinical questions and recommendations on the basis of the panel’s expertise and using a pragmatic approach based on both literature and clinical experience to provide support.

## Conclusions

The aim of this consensus was to point out practical recommendations (and not mandatory standard of practice) to support clinician’s decision-making in the management of SAH patients in hospitals with neurosurgical/neuroendovascular facilities after aneurysm securing. We provide 30 clinical recommendations aimed at supporting clinicians regarding the management of SAH patients in a center with neurosurgical/neuroendovascular facilities after the aneurysm treatment.

## Abbreviations

SAH: Subarachnoid hemorrhage
SIAARTI: Italian Society of Anesthesia and Intensive Care
ICU: Intensive care unit
DCI: Delayed cerebral ischemia
TCD: Transcranial Doppler
TCCD: Transcranial color Doppler
FVMCA: Flow velocity in the middle cerebral artery
CT: Computed tomography
CTA: Computed tomography angiography
DSA: Digital subtraction angiography
MRI: Magnetic resonance imaging
GCS: Glasgow coma scale
MAP: Mean arterial pressure
Hb: Hemoglobin
BCT: Body core temperature
ICP: Intracranial pressure
CPP: Cerebral perfusion pressure
AHA: American Heart Association
MCA: Middle cerebral artery
ICA: Internal carotid artery
ACA: Anterior cerebral artery
PTCA: Percutaneous transluminal balloon angioplasty
RBC: Red blood cell
NCS: Neurocritical Care Society
